# Postnatal mental health, breastfeeding beliefs, and breastfeeding practices in rural China

**DOI:** 10.1186/s13006-022-00504-6

**Published:** 2022-08-20

**Authors:** Qi Jiang, Evelyn Zhang, Nourya Cohen, Mika Ohtori, Sabrina Zhu, Yian Guo, Hannah Faith Johnstone, Sarah-Eve Dill, Huan Zhou, Scott D. Rozelle

**Affiliations:** 1 Freeman Spogli Institute for International Studies, Stanford University, Stanford, CA USA; 2 School of Public Health, University of California, Berkeley, Berkeley, CA USA; 3 West China School of Public Health and University, Chengdu, China

**Keywords:** Breastfeeding, Breastfeeding self-efficacy, Breastfeeding attitude, Mental health, Depression, Anxiety, Stress, Rural China

## Abstract

**Background:**

The importance of breastfeeding in low- and middle- income countries is well recognized, yet the importance of postnatal mental health on breastfeeding practices and beliefs in these settings has been understudied. This study investigates the associations between maternal mental health problems, breastfeeding beliefs and breastfeeding practices in rural China.

**Methods:**

Cross-sectional data were collected in November and December 2019 from 742 mothers of infants under 6 months old in rural Sichuan Province, China. Maternal mental health (depression, anxiety, and stress symptoms) was assessed using the Depression, Anxiety, and Stress Scale (short form). Breastfeeding beliefs were assessed using the Iowa Infant Feeding Attitude Scale and Breastfeeding Self-Efficacy Scale (short form). Breastfeeding practices were assessed through a 24-h dietary recall questionnaire. Ordinary least squares regression, multiple logistic regression and heterogeneous effects analyses were used to identify associations between symptoms of mental health problems and breastfeeding outcomes.

**Results:**

The average age of sample infants was 2.7 months. Among mothers, 13% showed symptoms of depression, 16% anxiety, and 9% stress. The prevalence of exclusive breastfeeding in the previous 24 h was 38.0%. Depression symptoms were significantly associated with breastfeeding attitude (𝛽= − 1.11, 95% CI: − 2.07, − 0.14) and breastfeeding self-efficacy (𝛽= − 3.19, 95% CI: − 4.93, − 1.45). Anxiety and stress symptoms were significantly associated with breastfeeding self-efficacy (𝛽= − 1.81, 95% CI: − 3.43, − 0.18 and 𝛽 = − 2.88, 95% CI: − 4.98, − 0.78, respectively). There were no significant associations between symptoms of mental health problems and exclusive breastfeeding. The heterogeneous effects analyses revealed that less educated mothers with symptoms of stress had lower odds of exclusive breastfeeding than educated mothers without symptoms of stress (OR: 0.53, 95% CI: 0.25,1.10). Mothers of younger infants had higher odds of exclusive breastfeeding than the mother of older infants, regardless of depression, anxiety, or stress symptoms.

**Conclusion:**

Symptoms of maternal mental health problems are significantly associated with breastfeeding attitude and self-efficacy; however, these symptoms are not associated with breastfeeding practices. Maternal educational level and infant age may play a role in mothers’ breastfeeding practices. To improve breastfeeding practices, interventions should employ a multi-dimensional approach that focuses on improving maternal mental well-being and considers demographic characteristics.

## Background

The international literature has shown that postnatal mental health problems among mothers can present a barrier to optimal breastfeeding [1–8]. Breastfeeding requires mothers to be engaged in and willing to participate in the feeding process; for mothers facing mental health problems, these demands can feel burdensome and ultimately discourage breastfeeding. Studies have found that depressed mothers are less likely to exclusively breastfeed and more likely to terminate breastfeeding earlier [1–7]. Similarly, studies have found that postpartum maternal anxiety is negatively associated with breastfeeding initiation, duration, and exclusivity [7, 9]. However, this research has mainly been conducted in high-income countries, and low- and- middle income countries (LMICs) remain understudied.

Postnatal mental health problems can also worsen breastfeeding beliefs, which have been found to be critical for breastfeeding practices. Existing global literature suggests that maternal depression affects breastfeeding beliefs, such as self-efficacy, which is defined as a mother’s confidence in her ability to breastfeed her newborn, and attitude, which is defined as a mother’s viewpoint and stance on breastfeeding. Maternal depression and anxiety have been found to be associated with lower breastfeeding self-efficacy [9–11]. Maternal depression and stress have also been shown to be associated with less favorable attitudes towards breastfeeding [12, 13], such as being more skeptical or unsupportive of the practice [8]. Having a positive attitude about breastfeeding is important since breastfeeding beliefs have been shown to not only be associated with higher rates of exclusive breastfeeding [8, 14–16], but also other measures of breastfeeding outcomes, such as the duration the infant is breastfed [15–17], and the mother’s satisfaction with breastfeeding [18].

Increasing evidence shows that the prevalence of postnatal mental health problems in low- and middle-income countries (LMICs), including China, is particularly high [19]. Given the association between mental health and breastfeeding, there is a concern that breastfeeding outcomes in these countries are being severely impacted. Like other LMICs, rural China has been shown to have a high prevalence of maternal mental health issues, as well as poor infant feeding practices. Postnatal depression rates have empirically been shown to range from 20 to 25% [20, 21], which are high compared to the global rate of 13% [22]. This high prevalence of maternal mental health problems is accompanied by low rates of exclusive breastfeeding, ranging from 4.2 to 28.7% [23–25]. These rates are low compared to the global rate of exclusive breastfeeding among infants younger than 6 months, which is 45.7% [26]. Yet, there have been no studies on the relationship between postnatal mental health and breastfeeding beliefs and practices in rural China. Considering the high prevalence of maternal mental health issues and poor infant feeding practices, the relationship between maternal mental health and breastfeeding outcomes in rural China needs to be better understood.

The goal of this study is to investigate the association between symptoms of postnatal mental health problems (depression, anxiety and stress sypmtoms), breastfeeding beliefs (attitudes and self-efficacy), and breastfeeding practices in rural China. Specifically, we pursue three objectives. First, we describe the prevalence of symptoms of mental health problems among new mothers in rural China and identify their breastfeeding practices and beliefs. Second, we examine the correlation between symptoms of postnatal mental health problems and breastfeeding beliefs and practices. Finally, we examine the heterogeneity in correlations between symptoms of postnatal mental health problems and breastfeeding beliefs and practices by the demographic characteristics of mothers.

## Methods

A cross-sectional study was conducted in rural areas of one prefecture in Sichuan Province. According to the National Bureau of Statistics of China, 48% of Sichuan’s population are rural residents. The average per capita disposable income in the rural areas of Sichuan is 13,331 RMB (1906 US dollars), far lower than the national average of 28,228 RMB (4033 US dollars) [27]. The study area can be considered relatively representative of rural southwestern China.

### Sampling

The study sample was drawn from the baseline sample of an interventional study assessing the impacts of a community health worker program on the health and nutrition of pregnant women, new mothers, and infants. The sampling strategy for this study was determined by power requirements to detect impacts of the intervention on infant hemoglobin concentrations, exclusive breastfeeding among infants under 6 months, and dietary diversity among infants older than 6 months (not reported in this paper). Using parameters from previous research, we assumed an intra-cluster correlation of .01 and a cluster size of 12 samples per township. Based on this, we calculated an overall sample frame of 80 townships with an average of 12 mother-child dyads per township.

The research team then implemented a 3-step sampling protocol to select households for the study. First, from the nine counties within the sample prefecture, the four nationally-designated “poverty counties” (pinkun xian) were included, which are counties that have more than 2% of their population living under the national poverty line of 3000 RMB (458 US dollars) [28]. These counties were selected in order to sample the mental health and breastfeeding outcomes of low-income rural mothers. Second, sample townships were chosen within each sample county. The sampling frame excluded non-rural townships and rural townships with populations less than 10,000. Of the remaining townships, 20 townships per county were randomly selected, resulting in a total of 80 rural townships. Third, the research team obtained a list of all households with pregnant women beyond their second trimester or with infants under 6 months old from the local county-level Maternal and Child Hospital in each sample county and selected a maximum of 25 eligible mother-child dyads per township. In townships with less than 25 eligible samples, the research team traveled to surrounding villages to enroll eligible participants until all eligible samples had been enrolled or a maximum of 25 samples was reached. In townships with more than 25 eligible samples, participants were randomly selected for enrollment until the maximum of 25 was reached.

Following this strategy, 1296 participants were sampled and 1233 agreed to participate in the survey. Because this study focuses on postnatal mental health and breastfeeding, the analytical sample excluded 353 pregnant women, 41 non-mother caregivers and 89 non-breastfeeding mothers. Eight respondents did not complete the survey and were also excluded. This resulted in a final sample of 742 breastfeeding mothers in 80 rural townships.

### Data collection

Data were collected from November to December 2019 by trained survey enumerators. During the training, enumerators were taught how to implement the survey instruments for each of the study’s main components. Once the training was completed, enumerators conducted one-on-one survey interviews at each sampled household. The survey collected data on postnatal mental health (symptoms of depression, anxiety, and stress), breastfeeding beliefs (attitudes and self-efficacy), breastfeeding practices, and demographic characteristics.

### Postnatal mental health

To measure the mental health of sample mothers, the research team used the Depression, Anxiety, and Stress Scale-21 (DASS-21). This is a 21-item short-form version of the DASS-42 that was originally created by Lovibond and Lovibond [29]. The DASS-21 scale has been validated in China [30, 31]. To complete the DASS-21, enumerators asked mothers to rank each statement (7 for each subsection) from 0 to 3, depending on how much the statement applied to them in the past week. Each individual’s DASS-21 score was then calculated by adding up the ranking for every sub-question in a specific section and multiplying the sum by 2. Hence, for each section, the total sum score could range from 0 to 42. Mothers who scored greater than or equal to 9 for depression, 7 for anxiety, and 14 for stress were considered to have symptoms of each respective mental health problem. It is important to note that the resulting score of the DASS-21 scale is not a clinical diagnosis but only a reasonable measurement of the severity of depression, anxiety, and stress symptoms.

### Breastfeeding beliefs

Two measures for breastfeeding beliefs were collected: breastfeeding attitudes and breastfeeding self-efficacy. To measure breastfeeding attitudes, enumerators administered the Iowa Infant Feeding Attitude Scale [32], which has been used in Turkey [12], Ethiopia [33], the United Kingdom [34], Russia [34], and the United States [35]. It has also been validated in China [36]. Breastfeeding mothers were read 18 statements, such as “breastfeeding is more convenient than formula feeding” and asked to use a 5-point Likert scale to express if they agreed or disagreed with the statement (1 being strongly disagree and 5 being strongly agree). Total scores range from 18 to 90. Statements that were representative of worse attitudes were reverse coded so that a higher score correlates to a more positive attitude about breastfeeding.

Mothers’ breastfeeding self-efficacy was assessed using the short form of the Breastfeeding Self-Efficacy Scale [37]. It has previously been used in Kenya [38], Turkey [39], and Malaysia [40], and was validated in Chinese by Ip et al. [41]. Breastfeeding mothers were asked to rate how confident they were about 16 breastfeeding-related items (e.g., “I manage to keep up with my infant’s breastfeeding demands”) using a 5-point Likert scale with 1 being not at all confident and 5 being always confident. Total scores range from 16 to 80, with higher scores corresponding to higher levels of self-efficacy.

### Breastfeeding practices

Breastfeeding practices were determined through a 24-h dietary recall questionnaire, in which enumerators asked mothers to list all the foods and liquids they fed their infant in the last 24 h. Based on this data, mothers were then divided into two feeding categories: exclusive breastfeeding and non-exclusive breastfeeding. As defined by the WHO, exclusive breastfeeding is breastfeeding while giving no other foods or liquids [42]. Non-exclusive breastfeeding in our study refers to any other feeding practices than exclusive breastfeeding, including predominant breastfeeding (feeding breastmilk along with other water or water-based liquids), and mixed breastfeeding (feeding breastmilk along with formula or animal milk, other liquids or solids).

### Demographic characteristics

Data on demographic characteristics of each sample family and infant were also collected by enumerators. Family characteristics included mother’s age, mother’s and father’s education levels, and family yearly income. Demographic characteristics of the infant were collected from the birth certificate, and included the infant’s gender, age in months, whether the infant had low birth weight, and whether the infant was born prematurely.

### Statistical analysis

The statistical analysis for the study is composed of four parts. First, the research team calculated the overall prevalence of maternal depression, anxiety, and stress symptoms, as well as the overall prevalence of breastfeeding outcomes, which include breastfeeding practice and beliefs. Second, an adjusted ordinary least squares (OLS) regression was used to identify the correlations between mental health and breastfeeding beliefs, while controlling for other potential confounders. The specification of the adjusted model is:

1 $${\mathrm{Belief}}_{\mathrm i}={\mathrm\beta}_0+{\mathrm\beta}_1\mathrm{Mental}\_{\mathrm{health}}_{\mathrm i}+{\mathrm{Control}}_{\mathrm i}+{\mathrm\varepsilon}_{\mathrm i}$$

where Belief_i_ refers to breastfeeding beliefs (breastfeeding attitude or breastfeeding self-efficacy). Mental _ health_i_ is the dummy variable for mental health (depression, anxiety, or stress symptoms), which takes the value of 1 when mother_i_ showed symptoms of mental health problems and takes the value of 0 when mother_i_ did not. Control_i_ is a set of control variables, including infant gender and age, whether the infant was born prematurely, whether the infant has low birth weight, maternal age and education level, paternal education level, and family income.

Third, a multivariate logistic regression was run to examine the associations between mental health problems and breastfeeding practices. The specification of the regression model is:

2 $${\mathrm{Practice}}_{\mathrm{i}}={\mathrm\beta}_0+{\mathrm\beta}_1\mathrm{Mental}\_{\mathrm{health}}_{\mathrm{i}}+{\mathrm{Control}}_{\mathrm{i}}+{\mathrm{\upepsilon}}_{\mathrm{i}}$$

where Practice_i_ takes the value of 1 when mother_i_ was fully breastfeeding and takes the value of 0 when mother_i_ was mixed breastfeeding. In Eq. (2), the definitions of the variables, Mental _ health_i_ and Control_i_ are the same as those in Eq. (1).

Finally, a heterogeneous analysis was conducted to measure the effects of symptoms of maternal mental health problems on breastfeeding practice by five demographic characteristics, including three socio-economic status (SES) characteristics (education levels of each parent and family income), maternal age, and infant age. The specification of the adjusted model is:

3 $${\mathrm{Practice}}_{\mathrm{i}}={\mathrm\beta}_0+{\mathrm\beta}_1\mathrm{Mp}\_{\mathrm{low}}_{\mathrm{i}}+{\mathrm\beta}_2\mathrm{Mp}\_{\mathrm{high}}_{\mathrm{i}}+{\mathrm\beta}_3\mathrm{Mh}\_{\mathrm{low}}_{\mathrm{i}}+{\mathrm{Control}}_{\mathrm{i}}+{\mathrm{\upepsilon}}_{\mathrm{i}}$$

where Mp _ low_i_, Mp _ high_i_, and Mh _ low_i_ are three binary variables. Mp _ low_i_ takes the value of 1 when mother_i_ shows symptoms of mental health problems and is of low SES/low maternal age/low infant age, and 0 otherwise. Mp _ high_i_ takes the value of 1 when mother_i_ shows symptoms of mental health problems and is of high SES/high maternal age/high infant age, and 0 otherwise. Mh _ low_i_ takes the value of 1 when mother_i_ does not have symptoms of mental health problems and is of low SES/low maternal age/low infant age, and 0 otherwise. Practice_i_ and Control_i_ are the same as those in Eq. (1).

## Results

Table 1 shows the demographic characteristics of the study sample. Of the infants sampled, 416 (56.1%) were male and the average age was 2.7 months. Only 22 sample infants (3.0%) were born prematurely, and 22 (3.0%) had low birth weight. The average age of the 742 mothers sampled was approximately 27.9 years old. Mothers were slightly less educated than fathers, with 297 mothers (40.0%) having graduated from high school compared to 341 fathers (46.0%).

**Table 1:** Descriptive statistics of demographic characteristics (*n* = 742)

	N (%) or Mean (SD)
Infant gender (% male), N (%)	416 (56.1%)
Infant age (months), mean (SD)	2.7 (2.0)
Premature, N (%)	22 (3.0%)
Low birth weight, N (%)	22 (3.0%)
Maternal age (years), mean (SD)	27.9 (4.9)
Mother graduated high school, N (%)	297 (40.0%)
Father graduated high school, N (%)	341 (46.0%)
Family yearly income (10,000 yuan), mean (SD)	7.4 (6.2)

The prevalence of symptoms of mental health issues and descriptive data on breastfeeding practices and beliefs among sample mothers is shown in Table 2. According to the data, 96 mothers (13.0%) exhibited symptoms of depression, 119 (16.0%) exhibited symptoms of anxiety, and 67 (9.0%) exhibited symptoms of stress. A number of 96 mothers exclusively breastfed their infants, which accounts for 38.0% of the sample. For breastfeeding attitude, mothers scored an average of 61.3, with a range of 18–90. For breastfeeding self-efficacy, mothers scored an average of 56.1, with a range of 16–80.

**Table 2:** Descriptive statistics of breastfeeding practices, knowledge, and beliefs (*n* = 742)

	N (%) or Mean (SD)
**Panel A. Postnatal mental health**	
Symptoms of depression in previous week, N (%)	96 (13.0%)
Symptoms of anxiety in previous week, N (%)	119 (16.0%)
Symptoms of stress in previous week, N (%)	67 (9.0%)
**Panel B. Breastfeeding practice**	
Exclusive breastfeeding in previous 24 h, N (%)	282 (38.0%)
Any breastfeeding in previous 24 h, N (%)	460 (62.0%)
**Panel C. Breastfeeding beliefs**	
Breastfeeding attitude score (range: 18–90), mean (SD)	61.3 (4.5)
Breastfeeding self-efficacy score (range: 16–80), mean (SD)	56.1 (8.8)

The OLS regression analysis for the correlations between mental health problems and breastfeeding beliefs are shown in Table 3. Mothers with symptoms of depression had significantly lower breastfeeding attitude scores (𝛽= − 1.11, 95% CI: − 2.07, − 0.14) and lower breastfeeding self-efficacy scores (𝛽= − 3.19, 95% CI: − 4.93, − 1.45). Additionally, mothers with symptoms of anxiety and symptoms of stress also had significantly lower breastfeeding self-efficacy scores (𝛽= − 1.81, 95% CI: − 3.43, − 0.18 for anxiety symptoms, and 𝛽 = − 2.88, 95% CI: − 4.98, − 0.78 for stress symptoms).

**Table 3:** Associations between postnatal mental health problems and breastfeeding beliefs (*n* = 742)

	Breastfeeding attitude		Breastfeeding self-efficacy	
	𝛽 (95% CI)	*p*-value	𝛽 (95% CI)	*p*-value
Symptoms of depression	−1.11 (−2.07, −0.14)	0.024	−3.19 (−4.93, − 1.45)	0.000
Symptoms of anxiety	−0.46 (− 1.36, 0.44)	0.314	− 1.81 (− 3.43, − 0.18)	0.029
Symptoms of stress	−0.70 (− 1.86, 0.46)	0.237	−2.88 (− 4.98, − 0.78)	0.007

Controlled for demographic characteristics and breastfeeding practices (infant gender, infant age, premature birth status, whether the infant was born with a low birth weight, maternal age, maternal education level, paternal education level, family income, and breastfeeding practice)

Table 4 reports the logit regression analysis of the associations between mental health problems and breastfeeding practices. None of the mental health problems measured were significantly associated with the mother’s breastfeeding practices. In order to determine if the associations between mental health and breastfeeding practices differed among various subgroups of the sample, a heterogeneous effects analysis was performed. The results are presented in Table 5, where each panel shows adjusted odds ratio of a mother who practiced exclusive breastfeeding given a specific demographic variable and whether or not she had symptoms of depression, anxiety, or stress.

**Table 4:** Associations between postnatal mental health problems and breastfeeding practices (*n* = 742)

	Adjusted OR (95% CI)	*P*-value
Symptoms of depression	1.12 (0.71, 1.76)	0.641
Symptoms of anxiety	1.17 (0.77, 1.79)	0.465
Symptoms of stress	0.84 (0.48, 1.46)	0.527

Mixed breastfeeding used as reference group. Controlled for demographic characteristics (infant gender, infant age, premature birth status, whether the infant was born with a low birth weight, maternal age, maternal education level, paternal education level, and family income)

**Table 5:** Heterogeneous effects of postnatal mental health problems on breastfeeding practices (*n* = 742)

	Symptoms of depression	Symptoms of anxiety	Symptoms of stress
Panel A: Maternal education level (reference group: without MH problem & high education level)			
(6) with MH problem & low education level	0.90 (0.49, 1.67)	0.88 (0.50, 1.59)	0.526* (0.25, 1.10)
(7) with MH problem & high education level	0.80 (0.39, 1.65)	1.03 (0.54, 1.99)	1.13 (0.45, 2.86)
(8) without MH problem & low education level	0.63** (0.43, 0.91)	0.65** (0.44, 0.95)	0.70* (0.49, 1.01)
(9) *p*-value of test (6) = (7)	0.777	0.701	0.182
(10) *p*-value of test (6) = (8)	0.213	0.261	0.418
Panel B: Family income (reference group: without MH problem & high family income)			
(1) with MH problem & low family income	1.27 (0.72, 2.25)	1.35 (0.76, 2.38)	1.10 (0.56, 2.17)
(2) with MH problem & high family income	0.89 (0.40, 1.95)	1.05 (0.55, 2.00)	0.48 (0.17, 1.39)
(3) without MH problem & low family income	0.98 (0.69, 1.38)	0.99 (0.69, 1.40)	0.97 (0.69, 1.36)
(4) *p*-value of test (1) = (2)	0.437	0.528	0.177
(5) *p*-value of test (1) = (3)	0350	0.267	0.710
Panel C: Maternal age (reference group: without MH problem & high maternal age)			
(11) with MH problem & low maternal age	1.35 (0.76, 2.38)	1.50 (0.90, 2.51)	1.16 (0.60, 2.21)
(12) with MH problem & high maternal age	1.11 (0.46, 2.69)	0.71 (0.26, 1.90)	0.44 (0.12, 1.68)
(13) without MH problem & low maternal age	1.17 (0.82, 1.66)	1.08 (0.76, 1.53)	1.13 (0.80, 1.59)
(14) *p*-value of test (11) = (12)	0.698	0.157	0.187
(15) *p*-value of test (11) = (13)	0.597	0.167	0.931
Panel D: Paternal education level (reference group: without MH problem & high education level)			
(16) with MH problem & low education level	0.91 (0.46, 1.79)	1.12 (0.61, 2.04)	0.64 (0.29, 1.40)
(17) with MH problem & high education level	1.31 (0.66, 2.62)	1.36 (0.70, 2.63)	1.29 (0.53, 3.16)
(18) without MH problem & low education level	1.09 (0.77, 1.54)	1.06 (0.75, 1.52)	1.11 (0.79, 1.56)
(19) *p*-value of test (16) = (17)	0.429	0.643	0.230
(20) *p*-value of test (16) = (18)	0.594	0.878	0.163
Panel E: Infant age (reference group: without mental health & high infant age)			
(21) with MH problem & low infant age	2.01** (1.09, 3.72)	2.97*** (1.67, 5.29)	1.64 (0.78, 3.47)
(22) with MH problem & high infant age	1.96** (1.02, 3.77)	1.16 (0.62, 2.17)	1.25 (0.56, 2.77)
(23) without MH problem & low infant age	2.72*** (1.94, 3.80)	2.34*** (1.67, 3.29)	2.52*** (1.82, 3.48)
(24) *p*-value of test (21) = (22)	0.951	0.018	0.604
(25) *p*-value of test (21) = (23)	0.329	0.416	0.256

Values shown as adjusted OR (95% CI)

*MH* Mental health

*p < 0.05, **p < 0.01, ***p < 0.001

Panel A shows the heterogeneous effects of maternal education level. Less educated mothers with symptoms of stress had lower odds of exclusively breastfeeding than educated mothers without symptoms of stress (OR: 0.53, 95% CI: 0.25, 1.10). Of mothers without symptoms of mental health issues, infants of mothers with low education levels were also significantly less likely to be exclusively breastfed (OR: 0.63, 95% CI: 0.43, 0.91 for depressive symptoms; OR: 0.65, 95% CI: 0.44, 0.95 for anxiety symptoms; OR: 0.70, 95% CI: 0.49, 1.01 for stress symptoms). Panel B, C and D shows the heterogeneous effects of family income, maternal age, and paternal education level, respectively, which yield no significant associations in any subgroups.

Panel E shows the heterogeneous effects of infant age. Mothers of younger infants with symptoms of depression or anxiety were significantly more likely to practice full breastfeeding (OR: 2.01, 95% CI: 1.09, 3.72; OR: 2.97, 95% CI: 1.67, 5.29, respectively) compared to mothers who had older infants and no symptoms. Mothers of younger infants without mental health symptoms were also generally more likely to practice exclusive breastfeeding than mothers of older infants (OR: 2.72, 95% CI: 1.94, 3.80 for depressive symptoms; OR: 2.34, 95% CI: 1.67, 3.29 for anxiety symptoms; OR: 2.52, 95% CI: 1.82, 3.48 for stress symptoms). Among mothers with older infants, mothers with symptoms of depression were more likely to practice exclusive breastfeeding (OR:1.96, 95% CI: 1.02, 3.77). Among mothers with symptoms of anxiety, those with younger infants showed significantly greater odd of exclusively breastfeeding than those with younger infants (*p* = 0.018).

## Discussion

The goal of this study was to examine the association between maternal mental health problems and breastfeeding beliefs and practices. From the sample of 742 mothers, this study found significant associations between maternal mental health and breastfeeding beliefs. Symptoms of depression were negatively associated with breastfeeding attitude and self-efficacy. In addition, symptoms of anxiety and stress were negatively associated with self-efficacy. These results align with the international literature that has found that postnatal mental health can have a negative impact on breastfeeding beliefs [9–13].

The overall correlation between maternal mental health and breastfeeding practices was not significant, contradicting previous findings in the international literature [1–6]. The results did show nuanced correlations between mental health and SES, with stress associated with a lower likelihood of fully breastfeeding among high income mothers. In addition, various demographic characteristics (mother age, infant age, and paternal education) were significantly correlated with breastfeeding practices. While there are mixed results on the relationship between various demographic characteristics and breastfeeding practices in the global literature, these findings are supported by other studies in LMICs showing that mothers are more likely to breastfeed exclusively if they have higher education levels and when their infants are younger [43, 44].

While maternal education was not significantly correlated with fully breastfeeding, there was a significant association with lower paternal education level. These results seem to contradict previous findings that higher paternal education levels are positively associated with breastfeeding [45, 46]. One possible explanation specific to the context of rural China is that more educated fathers are more likely to out-migrate for work than less educated fathers, leaving the mother and infant behind. Previous studies have shown that paternal support promotes exclusive breastfeeding [47, 48], so it is possible that less educated fathers are more likely to be at home and provide the support the mother needs to fully breastfeed.

This study has limitations. First, the study only looks at cross-sectional data and is therefore unable to draw conclusions on the directionality or causality of the relationship between mental health and breastfeeding outcomes. In addition, the original sample was powered to detect impacts of an intervention on infant hemoglobin, exclusive breastfeeding among infants under 6 months, and dietary diversity among infants older than 6 months; however, it was not powered to assess correlations between mental health and breastfeeding, and it is possible that our study is under-powered to detect these associations. Finally, our assessment of mental health problems was based on a self-report assessment of symptoms rather than a professional diagnosis. Due to stigma against reporting symptoms of mental illness in LMICs, it is possible that the prevalence of depression, anxiety and stress symptoms are underestimated among women in the sample.

This study also has many strengths. This research fills a gap in the literature surrounding the associations between maternal mental health and breastfeeding beliefs and practices, specifically in rural China. Additionally, this study includes a large sample of women with newborns aged 0–6 months, a population which very few studies have focused on. Future studies could include multiple survey waves to determine directionality or perform mediation or interaction analysis between maternal mental health and breastfeeding. Further research should also investigate why the relationship between maternal mental health and breastfeeding beliefs is not associated with breastfeeding practices in this population.

## Conclusions

Given that only 38% of sample mothers were exclusively breastfeeding their infants under the age of the 6 months, this field of study requires more attention from policymakers and researchers. Symptoms of maternal mental health problems are significantly associated with breastfeeding attitude and self-efficacy yet are not associated with breastfeeding practices. Maternal educational level and infant age may play a role in the association of maternal mental health problems and mother’s breastfeeding practices. Interventions to improve breastfeeding outcomes in rural China should target women in these vulnerable groups and use a multi-dimensional approach to further address the complexities of breastfeeding.

## Data Availability

Datasets used in the current study are available from the corresponding author upon reasonable request.

## Abbreviations

DASS-21: Depression, Anxiety, and Stress Scale (short form)
LMICs: Low- and Middle-Income Countries
OLS: Ordinary Least Squares
SES: Socio-Economic Status
WHO: World Health Organization
